# Serum 25-Hydroxyvitamin D Concentrations Are Inversely Correlated with Hepatic Lipid Content in Male Collegiate Football Athletes

**DOI:** 10.3390/nu10070942

**Published:** 2018-07-21

**Authors:** Xiaomin Sun, Zhen-Bo Cao, Kumpei Tanisawa, Satomi Oshima, Mitsuru Higuchi

**Affiliations:** 1 School of Public Health, Xi’an Jiaotong University Health Science Center, Xi’an 710061, China; gzhtxiaomin@xjtu.edu.cn; 2 Global Health Institute, Xi’an Jiaotong University Health Science Center, Xi’an 710061, China; 3 Faculty of Sport Sciences, Waseda University, Tokorozawa, Saitama 359-1192, Japan; tanisawa@nibiohn.go.jp (K.T.); msatomi3939@yahoo.co.jp (S.O.); mhiguchi@waseda.jp (M.H.); 4 School of Kinesiology, Shanghai University of Sport, 399 Chang Hai Road, Shanghai 200438, China; 5 Department of Physical Activity Research, National Institutes of Biomedical Innovation, Health and Nutrition, Tokyo 162-8636, Japan

**Keywords:** abdominal body fat, American football, ectopic fat, rugby football, vitamin D

## Abstract

Lower serum 25-hydroxyvitamin D (25(OH)D) concentrations are associated with more weight and fat mass gain in adults in the general population, but it is unknown whether this is the case in collegiate football athletes with greater body weight. This study aimed to investigate associations of serum 25(OH)D concentrations with body fat and ectopic fat accumulation, and to determine which fat indicators are closely related to serum 25(OH)D in male collegiate football athletes. Thirty-four collegiate athletes aged 21 years were recruited. Serum 25(OH)D concentrations and the levels of visceral fat area (VFA), vastus lateralis intramyocellular lipid (IMCL), extramyocellular lipid (EMCL), and intrahepatic lipid (IHCL) were measured. Serum 25(OH)D concentrations were negatively associated with the IHCL values (*r* = −0.372, *p* = 0.030), and the relationship remained after adjustment for several factors (*r* = −0.378, *p* = 0.047). Additionally, multiple stepwise regression analysis of IHCL content as the dependent variable indicated that 25(OH)D concentrations were a stronger predictor of IHCL content (β = −0.363, *p* = 0.030) than % body fat and VO_2_peak_FFM_. Higher serum 25(OH)D concentrations are more closely related to lower IHCL content rather than any other fat indicators, suggesting that increasing serum 25(OH)D concentrations may have some effect that inhibits lipid accumulation in hepatic tissue, especially in heavy athletes.

## 1. Introduction

Obesity has long been officially recognized as a key factor for the incidence of cardiovascular diseases [1]. In addition to the lipid accumulation in adipose tissue, the accumulation of lipid in non-adipose tissues (such as liver and muscle), also known as ectopic lipid accumulation, also has been associated with the development of cardiovascular diseases [2].

Young collegiate athletes undertaking regular and intensive exercise have presumably been considered to have a very low risk of cardiovascular and metabolic diseases; however, the clustering of metabolic risk factors has been recognized, especially in young heavy athletes [3,4,5], who often intentionally try to gain weight by increasing energy intake, where weight gain is associated with increases in body fat content and in risks of cardiovascular diseases [6,7]. Guo et al. [3] found that professional athletes of strength sports in the heavier weight class usually have a higher prevalence of metabolic syndrome and its individual components, including central obesity. The same results were also observed in Japanese collegiate heavyweight Judo athletes and National Football League players [4,5].

Circulating 25-hydroxyvitamin D (25(OH)D) is the main circulating metabolite of vitamin D, as well as the form used for the assessment of vitamin D status. Over the last few decades, vitamin D receptors (VDRs) were also detected in many different tissues, including skeletal muscle, adipose tissue, cardiac muscle, and immune tissue [8]. Available data indicate that low circulating 25(OH)D concentrations are common in obesity, and negative relationships with body mass index (BMI), percent body fat (% body fat), waist circumference (WC), visceral fat area (VFA), and subcutaneous fat area (SFA) have been reported in adolescents, young and healthy middle-aged individuals, and old adults [9,10,11,12]. Numerous studies found that higher levels of body fat usually leads to lower 25(OH)D concentrations [13,14]. Recently, it was observed that vitamin D may inhibit lipid accumulation through VDRs or calcium irons [15,16]. Epidemiologic evidence indicates that higher serum 25(OH)D concentrations are associated with less weight and fat mass gain in the future [17], and vitamin D treatments could reduce hepatic triglyceride accumulation in mice [18,19].

However, limited evidence shows that this associations also exist in athletes [20]. Heller et al. [21] found that serum 25(OH)D concentrations were negatively associated with total body fat indicators, such as BMI and % body fat, even after controlling for sex in collegiate athletes. Additionally, Barcal et al. [22] found a negative relationship between serum 25(OH)D concentrations and % body fat in collegiate male wrestlers, regardless of seasonal changes. Recently, in addition to body fat tissue, circulating 25(OH)D concentrations have been negatively associated with ectopic lipid content in adults in the general population [23,24,25]. Although excessive ectopic fat accumulation, for example, increased skeletal muscle intramyocellular lipid (IMCL), has been independently associated with insulin resistance in obese and type 2 diabetes subjects, athletes who are highly insulin sensitive have similar muscle fat content to that observed in insulin-resistant subjects [26]. This metabolic conundrum has been termed “athletes’ paradox”. However, to date, the relationships between 25(OH)D concentrations and ectopic lipid are still unclear in collegiate football athletes.

Therefore, the primary aims of this study were to evaluate whether body fat or ectopic fat indicators are closely related to serum 25(OH)D concentrations in overweight male collegiate football athletes.

## 2. Materials and Methods

### Subjects

Thirty-four male collegiate athletes aged 21 (range, 20–21) years participated in the current study in summer (from June to July), which included 13 collegiate American football athletes (10 linemen, 3 backs) and 21 collegiate rugby football athletes (12 forwards, 9 backs). The levels of % body fat, SFA, VFA, and VO_2_peak of one subject in the rugby football group were unobtainable for personal reasons. One person’s dietary data was excluded from analysis because his total energy intake was more than 9000 kcal.

All athletes were members of the American and rugby football teams in the East Japan College Football Association division 1. All participants had >3 years of training experience playing their type of football and performed resistance exercise at least 5 times per weeks for about 2 h per day. All participants were in good health and free from chronic diseases. Athletes were excluded if they were regularly taking vitamin D supplements or used sunscreen. All participants provided written informed consent before study enrollment, and the Ethical Committee of Waseda University approved this study (ethical approval code: 2011-060; June 2013). The study was conducted in accordance with the Declaration of Helsinki.

## 3. Procedures

### 3.1. Anthropometric Measurements

Body weight was measured using an electronic scale (Inner Scan BC-600; Tanita Inc., Tokyo, Japan), whereas height was measured using a stadiometer (YL-65; YAGAMI Inc., Nagoya, Japan), with subjects wearing minimal clothing and no shoes. BMI was calculated using the body weight and height measurements. WC was measured at the umbilical region with an inelastic measuring tape at the end of normal expiration to the nearest 0.1 cm. Dual-energy X-ray absorptiometry (DXA) (Hologic QDT-4500, DXA Scanner; Hologic Inc., Waltham, MA, USA) was used to measure the % body fat. The subjects were asked to wear loose-fitting, light clothes without any metal objects and to lie in a supine position on the scanning table during the total body fat scan.

### 3.2. Magnetic Resonance Imaging and Spectroscopy

The VFA and SFA were measured by magnetic resonance imaging as described previously [12]. The imaging conditions included a T1-weighted spin-echo and axial plane sequence with a slice thickness of 10 mm, a repetition time (TR) of 140 milliseconds, and an echo time (TE) of 12.3 milliseconds. The cross-sectional areas of the VFA and SFA at the umbilical level were determined using image analysis software (Slice-o-matic 4.3 for Windows; Tomovision, Montreal, QC, Canada). The coefficient of variation for the cross-sectional area at the umbilical level was 0.4%.

Hepatic ^1^H magnetic resonance spectroscopy (MRS) was performed using a 1.5-T whole-body scanner (Signa 1.5 T; General Electric, Inc., Milwaukee, WI, USA) with an 8-channel body array coil. A single voxel (30 × 30 × 20 mm^3^) for spectroscopy was selected in the right lobe of the liver while avoiding major blood vessels. Voxel shimming was performed to optimize the homogeneity of the magnetic field. The proton spectra of the liver were acquired using the point-resolved spectroscopy technique (repetition time = 2500 milliseconds; echo time = 35 milliseconds; 64 measurements; 1024 sample points). Acquisition was synchronized to the respiratory cycle and triggered at the end of expiration. MRS data were quantified using LCModel version 6.3 (Stephen Provencher, Oakville, ON, Canada). Intrahepatic lipid (IHCL) content was defined as the signal intensities at 1.3 and 1.6 ppm relative to the signal intensity of water at 4.7 ppm. The coefficient of variation between measurements was 7.5%.

Vastus lateralis intramyocellular lipid (IMCL) and extramyocellular lipid (EMCL) were assessed by ^1^H-MRS with a flexible surface coil wrapped around the participant’s upper leg. Care was taken to avoid vascular structures and adipose tissue deposits within the voxel. The T1 magnetic resonance images were acquired by using a point-resolved spectroscopy sequence with the following acquisition parameters: repetition time 2000 milliseconds; echo time 35 milliseconds; field of view, 200 mm. The voxel volume was (20 × 20 × 20 mm^3^). The water signal was suppressed by using chemically selective saturation.

### 3.3. Analysis of Blood Samples

Blood samples were collected in Venoject-II AutoSep tubes (gel, 9 mL; Terumo Corporation, Tokyo, Japan) from an antecubital vein of the forearm between 0830 and 0930 h after a 12-h overnight fast and then centrifuged at 3000× *g* for 15 min at 4 °C. Serum samples were stored at −80 °C until the time of analysis. Serum enzymatic activities of aspartate aminotransferase (AST), alanine aminotransferase (ALT) and γ-glutamyl transferase (γ-GTP) were determined using the ultraviolet method (BML Inc., Tokyo, Japan), respectively. Serum 25(OH)D concentrations were measured in duplicate with commercially available enzyme-linked immunosorbent assay kits (25(OH)D; Immundiagnostik AG, Bensheim, Germany) according to the manufacturer’s instructions. The Control samples provided by the Immundiagnostik AG group were analyzed with each run for quality control. The intra-assay coefficient of variation was 8.9% for 25(OH)D.

### 3.4. Cardiorespiratory Fitness

Cardiorespiratory fitness was assessed using a cycle ergometer (Ergomedic 828E; Monark, Varberg, Sweden), and the peak oxygen uptake was quantified as VO_2_peak. The graded cycle exercise began at a workload of 45–90 W and was increased by 15 W/min until the subject could no longer maintain the required pedaling frequency of 60 rpm. Heart rate and ratings of perceived exertion were monitored each minute during exercise. Additional details were published elsewhere [12]. To correct for fat mass, the peak oxygen uptake per kilogram of fat free mass (VO_2_peak_FFM_) was calculated.

### 3.5. Dietary Intake

Information on total energy intake, carbohydrate, protein, fat, vitamin D and calcium intakes were analyzed using the computerized nutritional analysis system of the Food Frequency Questionnaire Based on Food Groups (Kenpakusha, Japan) [27].

### 3.6. Statistical Analysis

All statistical analyses were performed with SPSS software, version 22.0 (SPSS, Inc., Chicago, IL, USA). The Kolmogorov-Smirnov test was performed to assess the normality of data distribution. The Student *t* test (for normally distributed variables) or Mann-Whitney *U* test (for non-normally distributed variables) was used to evaluate the differences in these variables according to American football and rugby football groups. Pearson correlation coefficients were computed between serum 25(OH)D and body indicators. Linear multiple regression analysis was performed to assess the association of serum 25(OH)D with the fat indicators, adjusted for group, position and total energy intake. All measurements and calculated values are presented as mean ± standard deviation (for normally distributed variables) or median (interquartile range) (for non-normally distributed variables). The statistical significance level was set at *p* < 0.05.

## 4. Results

**Figure 1:**
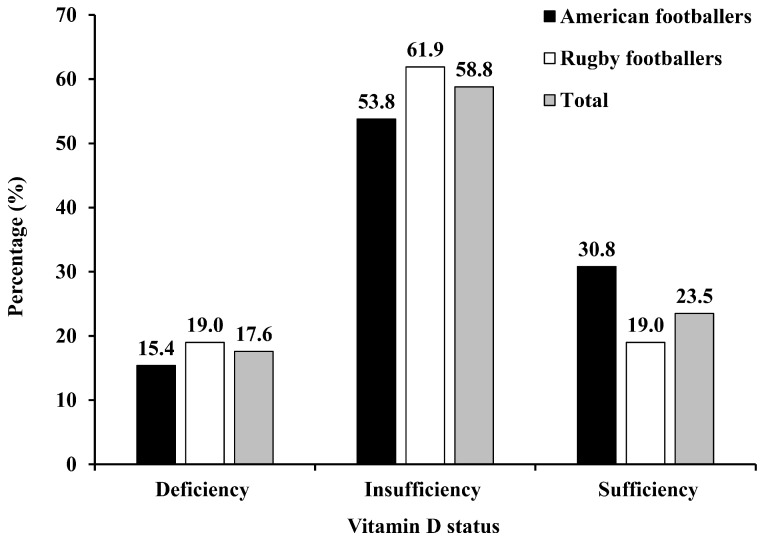
Prevalence of vitamin D deficiency and insufficiency.

Subject characteristics according to study groups are presented in Table 1 and Figure 1. The prevalence of vitamin D deficiency (<50 nmol/L) and vitamin D insufficiency (50–75 nmol/L) were 15.4% and 53.8% in American football collegiate athletes, and 19.0% and 61.9% in rugby football collegiate athletes, respectively. American collegiate football athletes had higher values of weight, WC, % body fat, and SFA than rugby football athletes (*p* < 0.05). No difference was observed in VO_2_peak_FFM_, IHCL, IMCL, EMCL, AST, ALT, γ-GTP, 25(OH)D, total energy, and other nutrients intake.

**Figure 2:**
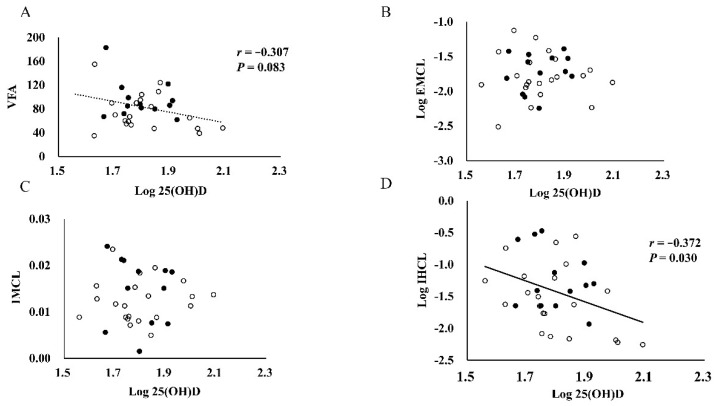
Associations among serum 25(OH)D with VFA (**A**), EMCL (**B**), IMCL (**C**), and IHCL (**D**). 25(OH)D, 25-hydroxyvitamin D; VFA, visceral fat area; IMCL, intramyocellular lipid; EMCL, extramyocellular lipid; IHCL, intrahepatic lipid. Closed and open circles represent data from American footballers and rugby footballers, respectively.

The correlations between serum 25(OH)D concentrations and body fat, ectopic lipid variables and other factors are showed in Table 2 and Figure 2A–D. Serum 25(OH)D concentrations were significantly and negatively correlated with the IHCL values (*r* = −0.372, *p* = 0.030). No significant associations were observed among serum 25(OH)D concentrations with IMCL, EMCL, and other variables (*p* > 0.05). After adjusting by study group, position, and total energy intake, the significant relationship between serum 25(OH)D concentrations and IHCL was still observed (*r* = −0.378, *p* = 0.047), whereas the other measures became insignificant (*p* > 0.05). In the subgroup analysis, serum 25(OH)D was observed to be negatively correlated with γ-GTP (*r* = −0.504, *p* = 0.033) and IHCL (*r* = −0.462, *p* = 0.053) in rugby football players after adjusting by total energy intake; whereas, no relationships were found in American football players. In addition, multiple stepwise regression analysis with IHCL content as the dependent variable indicated that the 25(OH)D concentrations was a stronger predictor of the IHCL content (β = −0.363, *p* = 0.030), independent of the % body fat and VO_2_peak_FFM_, and after adjustment for study group, position, and total energy intake.

## 5. Discussion

We investigated the relationships between serum 25(OH)D concentrations and body fat and ectopic fat in male collegiate football athletes. We first found that the serum 25(OH)D concentrations were inversely correlated with IHCL rather than traditional body fat indicators, suggesting increasing vitamin D may modulate the activity of liver adipocytes.

The prevalence of vitamin D deficiency (<50 nmol/L) and insufficiency (50–75 nmol/L) were 17.6% and 58.5%, respectively, in the present study, which is higher than the 25(OH)D concentrations in our previous study conducted in 81 adults in the general population (mean 65.4 vs. 35.3 nmol/L) [28]. Zhou et al. [29] also found obese men (BMI > 28 kg/m^2^) have relatively lower serum 25(OH)D concentrations in July (mean 46.1 vs. 65.4 nmol/L). Recently, our previous studies found that both acute exercise and long-term exercise training could increase serum 25(OH)D concentrations or prevent its seasonal reduction [30,31]. Football athletes have a regular exercise habit that could, in part, explain the relatively higher 25(OH)D concentrations in our study.

Since athletes perform a large amount of exercise training, they usually have been regarded as a healthy model with a lower risk of cardiovascular disease. However, according to recent studies, the heavy athletes are usually at significantly higher risk of cardiovascular diseases [3,4]. To enhance athletic performance, American and rugby football athletes usually have the intent to consume more energy to gain more weight [32], which was suggested to lead to lipid accumulation in adiposity and ectopic tissues [6,7]. Excessive lipid accumulation predisposes one to the development of cardiovascular diseases [2] and is negatively related to serum 25(OH)D concentrations [9,10,11,12]. In vitro, it has been suggested that vitamin D modulates the activity of adipocytes and may have effects on the inhibition of lipid accumulation through VDRs or calcium irons [15,16]. Prior evidence indicated that low circulating 25(OH)D concentrations were associated with future weight gain [17], and vitamin D treatment could significantly decrease hepatic triglyceride accumulation in obese and hyperglycemic mice [18,19].

Previous studies have showed that serum 25(OH)D concentrations were negatively correlated with BMI and % body fat, and with abdominal fat indicators in adults in the general population [9,10,11,12]. However, the relationships between 25(OH)D and fat variables were unclear in colligate football athletes. Heller et al. [21] found that serum 25(OH)D concentrations were negatively associated with BMI and % body fat in 42 male and female athletes who participate in several kinds of sports. A similar result was also observed in collegiate male wrestlers, and the negative relationship between serum 25(OH)D concentrations and % body fat was observed regardless of seasonal changes [22]. In the present study, correlation analyses showed that serum 25(OH)D concentrations were not related to any of the body fat variables, including BMI, % body fat, WC, and SFA in collegiate football athletes, except there was a trend of a negative correlation between serum 25(OH)D and VFA (*r* = −0.307, *p* = 0.083). According to our knowledge, few research studies have focused on the relationships between 25(OH)D and central body fat indicators in Asian football athletes. Although circulating 25(OH)D concentrations were negatively correlated with BMI and VFA in non-Asian adults, our previous study found that serum 25(OH)D concentrations were negatively correlated with VFA levels rather than BMI in Japanese adults [12]; Hao et al. [33] also found that the levels of VFA were the strongest predictors of serum 25(OH)D in Chinese men. The discrepancy of the relationships between 25(OH)D and these body fat indicators may be attributed to the different fat distribution in Asians and non-Asians [34]. Moreover, the relatively small sample size and narrow body fat range could be another potential explanation for the discrepancy.

In addition to body fat in adipose tissues, the relationships between fat accumulation in ectopic tissue and the incidence of cardiovascular disease have received more attention recently [2]. Prior evidence has showed that there are negative relationships between 25(OH)D concentrations and liver and muscle fat in adults in the general population [23,24,25]. However, this relationship has not been investigated in young collegiate football athletes. Our study found that there is a negative relationship between serum 25(OH)D concentrations and IHCL rather than IMCL and EMCL even after adjusting for several factors in collegiate football players. These disparate results of the associations of serum 25(OH)D concentrations with IMCL and EMCL may be partly related to “athletes’ paradox” [26]. The lipid droplets in muscle are usually located adjacent to the muscle mitochondria, which indicates that they function as a substrate energy source during exercise in athletes [35]. Exercise-trained athletes may have similar levels of IMCL with insulin-resistant obesity and type 2 diabetes mellitus [36]. In addition, we found that there was an inverse relationship between serum 25(OH)D concentrations and γ-GTP concentrations (*r* = −0.504, *p* = 0.033) in rugby players in the subgroup analysis. These data, including the finding from recent mice [18,19], suggest that increasing serum 25(OH)D concentrations may lead to the inhibition of hepatic lipid accumulation in overweight athletes.

The present study first found that higher serum 25(OH)D concentrations were closely related to the content of IHCL rather than traditional body fat indicators in male collegiate football athletes. However, a small sample size could be a limitation. Additionally, it is still unclear whether the results observed in our study could be extrapolated to other kinds of athletes. Finally, the cross-sectional nature of the study does not allow for determination of cause and effect. Longitudinal studies conducted on other kinds of sports are necessary to confirm the associations between serum 25(OH)D concentrations and fat tissue and ectopic fat indicators.

## 6. Conclusions

In conclusion, we observed that the prevalence of vitamin D deficiency and insufficiency was higher, but relatively higher than in adults in the general population. In addition, serum 25(OH)D concentrations were more closely associated with the intrahepatic cellular fat content than any of the indicators traditionally used to assess adiposity in overweight collegiate athletes.

Our results suggest that maintaining higher circulating 25(OH)D concentrations may lead to lower IHCL content, with the potential for subsequent reductions in cardiovascular disease risks in overweight athletes, and perhaps in others. These possibilities warrant testing in adequately designed randomized controlled trials.

## Figures and Tables

**Table 1:** Participant characteristics according to groups.

Variable	Total (*n* = 34)	American Football (*n* = 13)	Rugby Football (*n* = 21)
Age (years)	21.0 (20.0–21.0)	21.0 (20.0–22.0)	21.0 (20.0–21.0)
Height (cm)	175.0 ± 4.8	176.1 ± 4.5	174.4 ± 5.0
Weight (kg)	90.4 ± 10.9	95.5 ± 9.3	87.3 ± 10.8 *
BMI (kg/m^2^)	29.5 ± 3.0	30.7 ± 2.0	28.7 ± 3.3
WC (cm)	90.3 ± 8.0	94.8 ± 6.4	87.5 ± 7.8 *
% Body fat	16.6 ± 4.1	19.4 ± 2.6	14.8 ± 3.9 ^a,^*
SFA (cm^2^)	168.0 (89.5–222.0)	207.0 (170.0–275.0)	112.5 (72.3–183.0) ^a,^*
VFA (cm^2^)	82.8 ± 32.4	95.1 ± 31.6	74.8 ± 31.2 ^a^
VO_2_peak_FFM_ (mL/kg/min)	48.7 ± 5.6	46.5 ± 5.4	50.1 ± 5.4 ^a^
IHCL	0.037 (0.017–0.082)	0.047 (0.023–0.177)	0.024 (0.008–0.064)
IMCL	0.013 ± 0.006	0.014 ± 0.008	0.012 ± 0.005
EMCL	0.017 (0.012–0.030)	0.019 (0.012–0.032)	0.015 (0.012–0.028)
AST (IU/L)	24.5 (21.8–36.0)	25.0 (22.5–34.0)	24.0 (21.0–36.5)
ALT (IU/L)	34.1 ± 16.4	37.4 ± 20.8	32.1 ± 13.1
γ-GTP (IU/L)	28.0 (24.5–38.0)	29.0 (24.0–35.0)	27.0 (24.0–43.0)
25(OH)D (nmol/L)	61.3 (54.2–74.7)	62.3 (53.9–79.0)	60.3 (52.8–73.0)
Total energy intake (kcal)	3895.8 (3534.5–4365.0)	4071.7 (3662.8–5089.1)	3840.7 (3476.1–4232.5) ^b^
Carbohydrate intake (g)	511.6 (461.9–621.0)	522.6 (479.3–658.9)	501.0 (450.2–571.1) ^b^
Fat intake (g)	130.9 ± 35.0	143.0 ± 32.0	123.1 ± 35.3 ^b^
Protein intake (g)	143.2 ± 33.8	155.0 ± 37.5	135.5 ± 29.6 ^b^
Vitamin D intake (μg/day)	10.4 ± 4.1	11.2 ± 4.9	9.7 ± 12.4 ^b^
Calcium intake (mg/day)	1185.4 ± 442.0	1284.8 ± 402.3	1120.8 ± 464.3 ^b^

Data are presented as mean ± SD or median (IQR) values. BMI, body mass index; WC, waist circumference; SFA, subcutaneous fat area; VFA, visceral fat area; VO_2_peak, peak oxygen uptake; IHCL, intrahepatic lipid; IMCL, intramyocellular lipid; EMCL, extramyocellular lipid; AST, aspartate aminotransferase; ALT, alanine aminotransferase; γ-GTP, γ-glutamyl transferase; 25(OH)D, 25-hydroxyvitamin D. ^a^ *n* = 20; ^b^ *n* = 20, one person was excluded from the analysis because his total energy was >9000 kcal in rugby football; * *p* < 0.05 vs. American football players.

**Table 2:** Correlations between 25(OH)D concentrations and other variables.

Variables	25(OH)D		25(OH)D *	
	*r*	*p*-Value	*r*	*p*-Value
BMI (kg/m^2^)	−0.231	0.189	−0.012	0.952
WC (cm)	−0.279	0.110	−0.200	0.307
% Body fat	−0.036	0.841	0.164	0.405
SFA (cm^2^)	−0.138	0.444	−0.026	0.895
VFA (cm^2^)	−0.307	0.083	−0.218	0.265
IHCL	−0.372	0.030	−0.378	0.047
IMCL	0.032	0.860	−0.080	0.686
EMCL	0.019	0.913	0.125	0.526
ALT (IU/L)	−0.098	0.583	−0.048	0.806
AST (IU/L)	0.174	0.324	0.217	0.268
γ-GTP (IU/L)	−0.297	0.088	−0.317	0.100

Data are presented as Pearson’s coefficients. BMI, body mass index; WC, waist circumference; SFA, subcutaneous fat area; VFA, visceral fat area; IHCL, intrahepatic lipid; IMCL, intramyocellular lipid; EMCL, extramyocellular lipid; AST, aspartate aminotransferase; ALT, alanine aminotransferase; γ-GTP, γ-glutamyl transferase; 25(OH)D, 25-hydroxyvitamin D. 25(OH)D, IHCL, EMCL, AST, and γ-GTP were log-transformed; SFA was square root transformed for analysis. Bold font indicates significance (*p* < 0.05). * Data are adjusted by group, position, and total energy intake.
